# The Usefulness of Spectral Mammography in Surgical Planning of Breast Cancer Treatment—Analysis of 999 Patients with Primary Operable Breast Cancer

**DOI:** 10.3390/curroncol28040232

**Published:** 2021-07-12

**Authors:** Andrzej Lorek, Katarzyna Steinhof-Radwańska, Anna Barczyk-Gutkowska, Wojciech Zarębski, Piotr Paleń, Karol Szyluk, Joanna Lorek, Anna Grażyńska, Paweł Niemiec, Iwona Gisterek

**Affiliations:** 1 Department of Oncological Surgery, Prof. Kornel Gibiński Independent Public Central Clinical Hospital, Medical University of Silesia, 40-514 Katowice, Poland; wzarebski@sum.edu.pl; 2 Department of Radiology and Nuclear Medicine, Prof. Kornel Gibiński Independent Public Central Clinical Hospital, Medical University of Silesia, 40-514 Katowice, Poland; anna.barczyk@sum.edu.pl; 3 Department of Pathomorphology and Molecular Diagnostics, Prof. Kornel Gibiński Independent Public Central Clinical Hospital, Medical University of Silesia, 40-752 Katowice, Poland; ppalen@sum.edu.pl; 4 Department of Orthopaedic and Trauma Surgery, District Hospital of Orthopaedics and Trauma Surgery, 41-940 Piekary Śląskie, Poland; szyluk@urazowka.piekary.pl; 5 Department of Surgery, Ludwig Rydygier Hospital sp. z.o.o., 31-826 Kraków, Poland; asja.lorek@gmail.com; 6 Students’ Scientific Society, Department of Radiology and Nuclear Medicine, Medical University of Silesia, 40-514 Katowice, Poland; grazynska.anna@gmail.com; 7 Department of Biochemistry and Medical Genetics, School of Health Sciences, Medical University of Silesia, 40-752 Katowice, Poland; pniemiec@sum.edu.pl; 8 Department of Oncology and Radiotherapy, Prof. Kornel Gibiński Independent Public Central Clinical Hospital, Medical University of Silesia, 40-514 Katowice, Poland; igisterek@sum.edu.pl

**Keywords:** breast cancer, contrast-enhanced spectral mammography, mammography, comparative studies, pathology, surgery

## Abstract

Contrast-enhanced spectral mammography (CESM) is a promising, digital breast imaging method for planning surgeries. The study aimed at comparing digital mammography (MG) with CESM as predictive factors in visualizing multifocal-multicentric cancers (MFMCC) before determining the surgery extent. We analyzed 999 patients after breast cancer surgery to compare MG and CESM in terms of detecting MFMCC. Moreover, these procedures were assessed for their conformity with postoperative histopathology (HP), calculating their sensitivity and specificity. The question was which histopathological types of breast cancer were more frequently characterized by multifocality–multicentrality in comparable techniques as regards the general number of HP-identified cancers. The analysis involved the frequency of post-CESM changes in the extent of planned surgeries. In the present study, MG revealed 48 (4.80%) while CESM 170 (17.02%) MFMCC lesions, subsequently confirmed in HP. MG had MFMCC detecting sensitivity of 38.51%, specificity 99.01%, PPV (positive predictive value) 85.71%, and NPV (negative predictive value) 84.52%. The respective values for CESM were 87.63%, 94.90%, 80.57% and 96.95%. Moreover, no statistically significant differences were found between lobular and NST cancers (27.78% vs. 21.24%) regarding MFMCC. A treatment change was required by 20.00% of the patients from breast-conserving to mastectomy, upon visualizing MFMCC in CESM. In conclusion, mammography offers insufficient diagnostic sensitivity for detecting additional cancer foci. The high diagnostic sensitivity of CESM effectively assesses breast cancer multifocality/multicentrality and significantly changes the extent of planned surgeries. The multifocality/multicentrality concerned carcinoma, lobular and invasive carcinoma of no special type (NST) cancers with similar incidence rates, which requires further confirmation.

## 1. Introduction

Currently, breast cancer has the highest incidence rate of all cancers in women both in Poland and worldwide [1,2]. The choice between the surgical and systemic treatment method for breast cancer depends on the histological type and grading of carcinoma, the ER/PgR and Ki67 expression and the HER2 status, as well as on progression of the primary tumor and the axillary lymph nodes, the presence and extent of metastases in distant organs, the menopausal status, the age, physical condition, past, and concomitant diseases and the related treatment, as well as the patient’s preferences [3,4]. The choice between breast-conserving surgical treatment and mastectomy is, to a large extent, dependent on the size of the tumor and exclusion of the multifocality and multicentrality of cancer lesions [5,6]. Multifocal and multicentric breast cancers are defined as the presence of two or more tumors within the same breast—multifocal when they occur within one quadrant of the breast, and multicentric, when they occur in two or more quadrants [7,8]. In imaging diagnostics, multifocality is assumed to exist when the distance between the lesions is lower than or equal to 5 cm, while multicentrality—when the distance is higher than 5 cm [9]. Exploring the impact of additional neoplastic foci in the breast on the scope of surgical treatment for the present study, multifocal and multicentric cancers were commonly defined as multifocal-multicentric cancers (MFMCC).

From among the many imaging methods available, mammography is the most useful and most used option for detecting focal lesions in breast glands. Nowadays, digital mammography (MG) has almost completely superseded its analog counterpart, allowing for high-quality breast imaging with higher resolution, improved dynamics, as well as quick data and image processing. However, its overall sensitivity and specificity in detecting breast cancer remain at the level of 62–75% [10,11,12,13,14]. Therefore, comprehensive pre-operative imaging assessments, such as the first-line mammography, followed by methods of higher sensitivity and specificity, are essential in patients with suspected multiple breast cancer.

Contrast-enhanced spectral mammography (CESM) is based on the double-energy technology that capitalizes on the inherent difference in X-ray attenuation of breast tissue and iodine. It provides morphological information similarly to conventional mammography and additionally makes it possible to visualize breast areas that exhibit enhanced uptake of the contrast agent most commonly related to neoangiogenesis, as is the case with breast magnetic resonance imaging (MRI) [15]. On the other hand, CESM uses X-rays as does conventional mammography. The average glandular dose (AGD) for a low-energy image is equal to one conventional mammography, while for a high-energy image—it is approximately 20% of the dose from one conventional mammogram. As a result, the total radiation dose delivered during a single CESM corresponds to approximately 1.2 times the dose in standard digital mammography [16]. Recently, CESM has shown similar sensitivity and specificity compared to MRI in detecting breast lesions at lower cost, faster speed, greater patient compliance, especially when claustrophobic. In addition, it can effectively detect microcalcifications [17,18,19,20]. Our study aimed at analyzing the presence of additional cancer foci in breast cancer visualized in conventional digital MG and contrast-enhanced spectral mammography (CESM), compared with postoperative histopathological examination, as well as to compare the relevance of these imaging methods in planning the scope of surgical treatment.

## 2. Materials and Methods

### 2.1. Patients and Procedures

In this retrospective study, we analyzed medical records of patients with primary operable breast cancer. The surgeries were performed between January 2013 and May 2020 at the Department of Oncological Surgery, University Clinical Center of the Medical University of Silesia, in Katowice, Poland. The inclusion criteria for the study included: a tumor initially confirmed by core-needle biopsy, by the recommendations of the Polish Union of Oncology (by European standards), and a full set of diagnostic tests (digital mammography, spectral-contrast mammography with additional ultrasound examination of the breast, regional lymph nodes, abdomen, and chest X-ray). We excluded patients with significant post-biopsy changes (e.g., hemorrhage) affecting image quality. Those patients who had not received surgery for metastatic cancer at the time of the biopsy were also excluded. Finally, 999 patients were included in our analysis. 

All the subjects had an MG examination that was performed in our Mammography Laboratory as part of the screening test programs or outside our department. However, in the course of this analysis, each of the obtained MG results was reassessed by two of our specialists with a total work experience of over 20 years. Each patient who had been diagnosed with breast cancer in core-needle biopsy received additional (apart from the MG) CESM. All CESM examinations were performed in the Mammography Laboratory of our center and were re-assessed by one radiologist with 8 years of experience in CESM diagnostics. After completing the diagnostics, the final therapeutic decision was made based on an arrangement of interdisciplinary case conferences of the Breast Cancer Unit with the participation of the patient and a team of specialists, including an oncological surgeon, a clinical oncologist, a radiotherapist, a radiologist, and a pathomorphologist. The patient was able to ask questions and expressed informed consent to the proposed treatment. After consultations the patients underwent surgeries. 

### 2.2. Imaging Modalities

Before performing CESM each patient had creatinine and GFR levels assessed also all the patients filled out a survey which was used as the basis for eliminating those with a risk of allergic reactions, potential pregnancy, and/or hyperthyroidism.

All CESM examinations were carried out with a digital mammography device dedicated to performing dual-energy CESM acquisitions (SenoBright, GE Healthcare, 3000 N. Grandview Blvd., Waukesha, WI, USA). An intravenous injection of 1.5 mL/kg of body mass of a non-ionic contrast agent was performed. The exposure pair (low and high energy) was performed automatically.

Specific image processing of low-energy and high-energy images was done to obtain subtraction images to highlight contrast enhancement and suppress structured noise due to fibroglandular breast tissue [21]. Rhodium anode material was used for all acquisitions, with molybdenum and rhodium filters with kVp ranging from 26 to 32 used for low energy acquisitions. The total duration of the examination was usually around 10 min. After examination, the patients were observed for approximately 30 min, for the occurrence of any adverse reactions.

The MG and CESM images were assessed according to the BI-RADS scale (Breast Imaging Reporting and Data System). A lesion whose cancerous nature had already been confirmed in core-needle biopsy was classified as BI-RADS 6, whereas additional foci suspected of multifocality or multicentrality of the cancerous process were classified as BI-RADS 4 or BI-RADS 5. On mammograms and subtraction images in CESM in the CC and MLO projections, three measurements were taken of the tumor classified as BI-RADS 6, and three measurements of the lesion suspected to contain MFMCC. Besides the measurements, the assessment was also performed for the lesions’ location, morphology (a nodule/an amorphous area of contrast enhancement—linear, segmentary, regional), and contrast enhancement. Next, the additionally identified lesions (suspected MFMCC) were visualized on second-look ultrasound or in MRI and subjected to core-needle biopsy (under USG or MRI). The statistical analysis included one (i.e., the biggest) dimension of the tumor.

**Figure 1:**
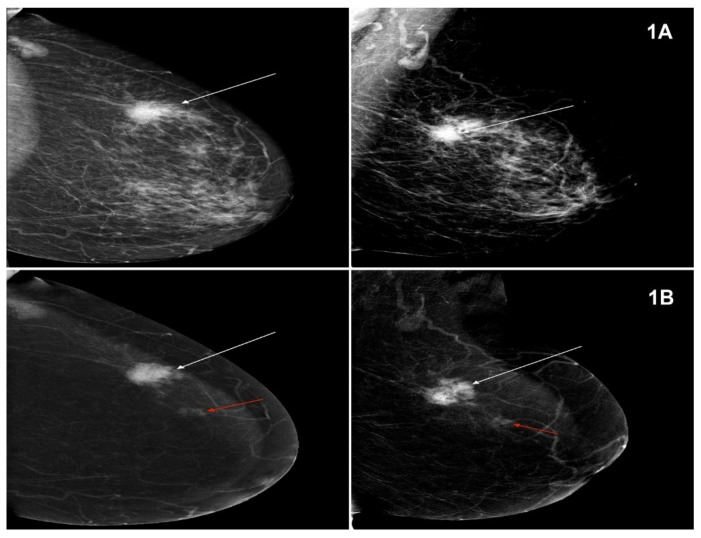
(**A**) Mammography (MG, low-energy images)—tumour in the left breast, in the upper external quadrant, measuring 35 mm—Breast Imaging Reporting and Data System (BI-RADS) 6 (white arrow). Projection: CC and MLO, HP-NST GIII. (**B**) Contrast-enhanced spectral mammography (CESM) (subtraction images)—tumour in the left breast, in the upper external quadrant, measuring 35 mm, enhanced upon contrast injection—BIRADS 6, HP: NST GIII (white arrow). A focus of amorphous contrast enhancement of a focal type, measuring 10 mm, at a distance of 25 mm from the tumour. BI-RADS 4 (red arrow). Projection: CC and MLO, HP-NST GII.

**Figure 2:**
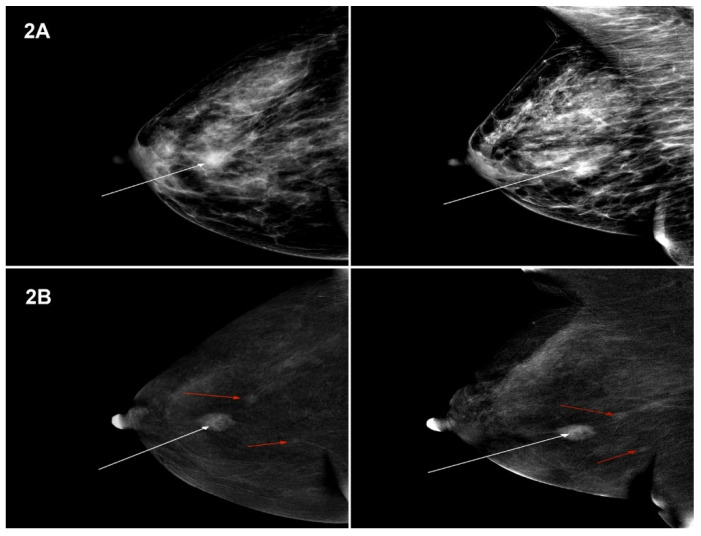
(**A**) MG (low-energy images)—tumour in the right breast, in the central part, measuring 16 mm—BI-RADS 6 (white arrow). Projection: CC and MLO, HP-Ca lobulare GIII. (**B**) CESM (subtraction images)—tumour in the right breast, in the upper external quadrant, measuring 15 mm, enhanced upon contrast injection—BIRADS 6, HP: Ca lobulare GIII (white arrow). Foci of amorphous contrast enhancement of a focal type, measuring 6 mm and 5 mm, at a distance of 12 mm and 26 mm from the tumour—BI-RADS 4 (red arrows). Projection: CC and MLO, HP-Ca lobulare GIII.

The lesions visualized in MG and CESM, defined according to the BI-RADS classification, were compared with the histopathological examination. Sample images of lesions in MG and CESM for NST and lobular cancer types (Figure 1A,B and Figure 2A,B).

### 2.3. Histopathological Examination

At the Department of Histopathology of the Medical University of Silesia, 2 experienced pathologists assessed the preparations. The T feature was defined based on the largest size of the primary tumor, and the N feature based on the number of metastatic lymph nodes. Tumors up to 2 cm were completely embedded in paraffin, and the larger 1microscopic evaluation of the lesions was performed using a microscope and Olimpus cellSens Dimension^®^ software (Japan). Next, they were marked in pairs with the ink of the same color and the individual layers were given numbers to allow for restoring the entire largest section of the tumor. The T dimension of the tumor was the sum of transverse measurements of the particular lesion parts. The tumors were defined as MFMCC if the distance between two lesions was separated by at least 5 mm of healthy tissue [22]. All the additional neoplastic foci diagnosed histopathologically had their histological features defined, including the tumor size, type, and malignancy level. The study included infiltrating cancers and in situ cancers.

### 2.4. Data Analysis and Statistical Method

The analysis included the results of 999 patients selected according to the aforementioned inclusion criteria. Patients’ age distribution was analyzed and tested for normality using the Kołmogorov–Smirnov test. The average, minimum and maximum values in the sample, as well as standard deviation, were determined for the variable studied. The subsequent part of the statistical analysis involved the construction of contingency tables for the results of MFMCC detectability for each of the diagnostic methods under analysis, compared with HP. The analysis of these tables served as the basis for calculating the values of sensitivity, specificity, negative predictive value (NPV), and positive predictive value (PPV) for each of the methods (MG and CESM). The 95% confidence intervals for the calculated sensitivity and specificity values were determined based on the Clopper–Pearson estimation method, using the Z test for a single proportion.

The significance limit for the calculations was established at *p* = 0.05 A quantitative summary was also prepared for the histopathological types of cancers, and the level of their detectability was determined for the diagnostic methods under analysis. The diagnostic results in the methods under analysis served as the basis for determining the rate of decision change in the treatment procedure. The data were analyzed using an Excel spreadsheet and Statistica software.

## 3. Results

Assessment of identification of multifocal-multicentral breast cancers (MFMCC) in digital MG and CESM is presented in Table 1. 

The comparative analysis showed that, in 779 (77.97%) of cases, MG and CESM both indicated that the changes were unifocal. Similar compliance in terms of MFMCC was revealed in 47 (4.70%) cases. In the remaining part of the assessments, there were discrepancies between the indications of the two methods. The 164 (16.41%) cases diagnosed as unifocal in MG were identified to contain MFMCC in CESM, while in the 9 (0.90%) cases under examination, the MG found the presence of MFMCC which was contrary to the results concerning unifocality in CESM. Due to these discrepancies, the HP results were adopted as a reference method and the level of compliance was examined between this method and the results obtained in mammography (MG) and CESM (Table 2). 

In MG, as far as the detection of MFMCC is concerned, the following findings were reached: true-positive results 48, false-positive results 8, false-negative results 146 and true-negative results 797. The examination sensitivity for discrimination of MFMCC from unifocal disease for MG was 24.74%, its specificity −99.01%, whereas the PPV (positive predictive value) was −85.71% and the NPV (negative predictive value) was −85.52%. The accuracy of MG was equal to −84.58%.

In CESM, as far as the detection of MFMCC is concerned, the following findings were made true-positive results 170, false-positive results 41, false-negative results 24 and true-negative results 764. For CESM, the sensitivity was 87.63%, its specificity, PPV, NPV, and accuracy were 94.91%, 80.57%, 96.95%, 93.49%, respectively.

A total of 999 surgical interventions were performed, involving the following procedures (Table 3).

The subsequent analysis focused on the histopathological types of cancers diagnosed as MFMCC in comparable mammography techniques in relation to the general number of cancers identified in HP (Table 4).

The analysis focused on the number of changes in the scope of conserving therapy into different types of mastectomy upon identification of MFMCC in CESM (Table 5). Based on the MG performed, the plan was to conduct 582 conserving procedures (WLE). After performing CESM and visualizing MFMCC in 116 cases, a decision was taken to conduct different types of mastectomy. The decision change rate was 116/582 = 19.9%. Among those 116 patients, the HP results confirmed MFMCC in 104 (89.6%) cases, and 12 (10.3%) cases were false-positive (the preoperative core-needle biopsy of those patients revealed atypical ductal hyperplasia). In the group of patients with MFMCC on conserving breast therapy, there were nine cases where the HP examination revealed positive margins (R1 resection), which required local radicalization.

## 4. Discussion

Multifocal cancers are defined as concomitant lesions, occurring within the same quadrant, with the distance between the lesions not exceeding 5 cm and the foci lying at least 5 mm from each other. Multicentral cancers, on the other hand, are located in different quadrants of the breast [23]. Multifocal and multicentral cancers (MFMCC) are correlated with more frequent lymph node involvement compared to unifocal cancers, and with more frequent infiltration beyond the nodal capsule, higher risk of local recurrence, and worse prognosis [24,25]. Identification of multifocality (MF) or multicentrality (MC) determines the method of surgical treatment for the patients.

The objective of our paper was to analyze the impact of spectral mammography on the scope of surgical treatment in women diagnosed with breast cancer. Therefore, multifocal and multicentral cancers were analyzed together. Depending on the source, multifocal/multicentral cancerous lesions in the breasts are described in approximately 5–12% of cases [26,27,28].

In our study, multifocality and multicentrality were confirmed in the histopathological examination in 194 (19.2%) of the subjects. The preponderance was composed of invasive infiltrating lobular breast cancer and no special type (NST) cancer. There was no statistically significant difference in the presence of multifocality/multicentrality between those two most common cancers: lobular and NST (27.78% vs. 21.24%, *p* = 0.22).

The sensitivity of digital MG in detecting multiple lesions in the breasts depends on their structure—others have shown that it decreases along with the increase in the glandular component. Depending on the sources, the sensitivity of MG in breasts with predominantly glandular tissue decreases significantly and ranged from 45% to approximately 60% [23,29].

In our analysis, the sensitivity of MG for detecting MFMCC amounted to 24.74%. During another assessment, the so-called second look, there was no significant increase in the sensitivity of MG. Similar experiences are presented by Bozzini- second look assessment of MG does not significantly increase the number of identified additional cancer foci [29].

The sensitivity of mammography for detecting the multifocality/multicentrality of the cancerous process is insufficient. Therefore, methods are being sought for reliable assessment of the staging of cancerous processes. It seems that this position today can be assumed by CESM.

CESM has higher sensitivity and specificity in identifying focal lesions in the breasts, especially in the case of highly glandular breasts, compared to standard MG [30]. Numerous studies have shown that the sensitivity of CESM in detecting breast cancer ranges from 92.7% to 100%. The specificity, on the other hand, is lower and ranges from 41% to 94% depending on the study [16,31,32,33,34]. A meta-analysis by Zhu et al. [35] showed a combined sensitivity of 89% (95% CI, 88–91) and specificity of (95% confidence interval (CI), 82–85) respectively. The NPV of CESM is also high, ranging from 92% to 100% [16,31,33,36] with a PPV greater than MRI (93–97%) [17,37]. 

CESM has comparable sensitivities and specificities to MRI examinations, yet at a lower cost, greater potential for access, and less time necessary to perform it, which makes CESM an attractive substitute for MRI. Both spectral mammography and magnetic resonance imaging are based on contrast enhancement of pathological foci, to visualize angiogenesis. Therefore, the discussion also refers to the publications that explore this issue in MR examination. Magnetic resonance imaging has been proven to have higher sensitivity in detecting neoplastic lesions than digital mammography and ultrasound [38,39]. Research shows that even 14–16% of tumors visible in MRI may remain invisible in MG, and multifocal tumors visualized in MRI and not diagnosed in mammography are often invasive cancers smaller than 10 mm [40,41]. Using MRI in the preoperative diagnostics of breast cancer patients results in modification of the treatment method in every fifth patient [41,42,43]. The decision change rate concerning the planned treatment in our study after CESM amounted to 20%.

In our study, CESM showed high sensitivity (87.63%) and specificity (94.91%) in detecting MFMCC. Jochelson et al. [37] in the pre-operative examination of breast cancer patients, showed that CESM was less sensitive in detecting additional ipsilateral neoplastic lesions compared to MRI (CESM detected 14/25 (56%) additional tumors, and MRI 22/25 (88%)). In addition, Lee-Felker et al. [17] in their study showed that CESM is as sensitive as MRI in detecting additional foci of disease with CEM identifying 11 of 11 (100%) of additional foci compared to 10 (91%) with MRI. Both studies found CESM had significantly fewer false-positive results and higher PPV compared to MRI. The authors suggest that CESM has the potential to be a useful additional imaging method in women with breast cancer when selecting an appropriate surgical method. In addition, they notice an additional advantage of CESM in the diagnosing and evaluating of asymptomatic women with dense breasts or an increased risk of cancer. In our analysis, CESM showed 41 false-positive results which is lower than in the multicentric study by COMICE, which analyzed this indicator in a group of patients after MRI [44]. It should be remembered that false-positive results expose patients to the unnecessary extension of the surgical procedure, which may be associated with an unsatisfactory cosmetic effect and a reduction in patients’ quality of life.

In our study, the multifocality/multicentrality of the neoplastic process also concerned NST and lobular cancers with similar prevalence, and the change of a decision about the scope of surgical interventions did not differ between those groups. These results require confirmation in further studies. If they are confirmed, a discussion must be launched on extending the indications to CESM before treatment in women diagnosed with breast cancer, regardless of the histopathological type.

In the present paper, we analyzed 999 diagnosed breast cancer patients who were initially operated on and subsequently had their diagnostics extended by CESM. To the best of our knowledge, the analysis of the relevance of spectral mammography in planning surgical treatment of breast cancer encompasses one of the largest groups. Additionally, it draws attention to the insufficient sensitivity of standard mammography in identifying the multifocality/multicentrality of the neoplastic process and the absence of a statistically significant difference in the multifocality/multicentrality of the neoplastic process between NST and lobular cancers.

To the best of our knowledge, few papers have been published analyzing the relevance of CESM in planning surgical treatment. While analyzing the diagnostic precision of CESM in suspected breast calcifications and its impact on the ensuing surgical decisions in a small group of 147 female patients, authors state that CESM only slightly improves the diagnostic precision in assessing breast calcifications and there is no significant value compared to MG in terms of planning the surgical treatment [45]. Our study analyzed all the lesions suspected of additional neoplastic foci (the analysis included tumors, focal asymmetries, calcifications) on a significantly larger group of 999 patients—among whom 116 (20%) had the surgical decision changed from a conserving therapy to mastectomy. Importantly, the chanced decisions concerned both lobular and NST cancers to almost the same extent.

Exact preoperative knowledge of the extent, size, and location of cancer lesions is the prerequisite for proper surgical intervention.

CESM led to a significant improvement in detecting MFMCC compared to MG and had a crucial influence on undertaking surgical decisions. Exact breast imaging and visualization of additional neoplastic foci may make it possible in the future to resign from post-operative breast radiotherapy after conserving treatment in a considerably larger group of patients. This will help to reduce the number of complications in female patients, and the treatment costs will be significantly lower.

CESM led to a significant improvement in detecting MFMCC compared to MG and had a crucial influence on surgical decisions. Exact breast imaging and visualization of additional neoplastic foci may make it possible in the future to resign from post-operative breast radiotherapy after conserving treatment in a considerably larger group of patients. This will help to reduce the number of complications in female patients, and the treatment costs will be significantly lower.

The limitation of our study is the fact that some MG examinations were performed outside our center, thus having a slightly lower technical quality, which might have resulted in worse visualization of additional foci in MG. In the entire study group of 999 operated patients observed to date, local recurrence was noted in four cases. Therefore, we think that exact advancement assessment has crucial meaning during qualification to treatment. Other limitations of our study were that it was a single-center study and all CESM examinations were conducted on a single vendor system. Our study also did not compare the effectiveness of CESM and MRI in detecting MFMCC. This limitation is because in our center we follow the European Society of Breast Cancer Specialists (EUSOMA) guidelines in the use of MRI. The MRI examination is currently recommended in the following clinical situations: a newly diagnosed lobular breast carcinoma confirmed by breast biopsy, patients with the genetically detected mutation, and patients under the age of 60 years who manifest a discrepancy of more than 1 cm in the tumor’s size between MG and US. Due to the above criteria, adding MRI to our analysis would significantly reduce the number of patients enrolled in the study our study, we wanted the largest possible group of patients with CESM performed to best define its effectiveness in detecting MFMCC. However, we plan to publish a study analyzing the MG, CESM, and MRI results shortly [46,47].

## 5. Conclusions

In conclusion, mammography offers insufficient diagnostic sensitivity for detecting additional cancer foci. The high diagnostic sensitivity of CESM effectively assesses breast cancer multifocality/multicentrality and significantly changes the extent of planned surgeries. There was no significant difference in the incidence of MFMCC in both lobular and invasive carcinoma of no special type (NST), which requires further confirmation.

## Figures and Tables

**Table 1:** The share of multifocal-multicentral breast cancers (MFMCC) in MG and CESM.

MG	CESM		Row in Total *n*
	Unifocal *n*	MFMCC *n*	
Unifocal	779	164	943
MFMCC	9	47	56
Total	788	211	999

**Table 2:** Compliance in identification of multifocal-multicentric breast cancers (MFMCC) in digital MG and CESM confirmed in the histopathological examination (HP).

Postoperative HP	MG		CESM		Method in Total *n*
	Unifocal *n*	MFMCC *n*	Unifocal *n*	MFMCC *n*	
Unifocal	797	8	764	41	805
MFMCC	146	48	24	170	194
Total	943	56	788	211	999

**Table 3:** Surgical procedures.

Surgical Procedure	*n*	%
Madden type radical mastectomy	310	31.03
Wide local excision (WLE) with sentinel lymph node biopsy (SLNB)	333	33.33
Wide local excision (WLE) with axillary lymph node dissection (ALND)	79	7.91
Total (simple) mastectomy	31	3.10
Total (simple) mastectomy with SLNB	185	18.52
Subcutaneous mastectomy with reconstruction with SLNB	6	0.60
Subcutaneous mastectomy with reconstruction with ALND	2	0.20
WLE	53	5.31

**Table 4:** Types of cancer in the post-operative histopathological examination (HP) diagnosed as multifocal-multicentric breast cancers (MFMCC) in MG and CESM.

HP	All Occurrences *n*	MG-MFMCC *n* (%)	CESM-MFMCC *n* (%)
NST (carcinoma of no special type)	631	37 (5.86)	134 (21.24)
Infiltrating lobular cancer	144	11 (7.64)	40 (27.78)
Special subtypes	75	4 (5.33)	16 (21.33)
DCIS HG	70	0 (0.00)	9 (12.86)
DCIS LG	29	1 (3.45)	2 (6.90)
LCIS (pleomorphic subtype)	4	0 (0.00)	0 (0.00)
Infiltrating ductolobular cancer	46	3 (6.52)	10 (21.74)
In total	999	56 (5.61)	211 (21.12)

**Table 5:** A change in the scope of procedures upon identification of multifocal-multicentric breast cancers (MFMCC) in CESM.

Types of Conserving Procedures	Surgeries Planned on the Basis of MG *n*	Surgeries Performed upon Visualisation of MFMCC in CESM *n*	Number of Changes in the Scope of Procedure into Difference Kinds of Mastectomy *n* (%)	Local Radicalisation in the Group of Patients on Conserving Treatment with MFMCC *n*
WLE + ALND	124	79	45 (7.7)	2
WLE	57	53	4 (0.68)	1
WLE + SLNB	401	333	68 (11.6)	6
In total	582	465	116 (19.9)	9

## Data Availability

Data is available upon special request.
